# Early weight measures and long-term neuropsychological outcome of critically ill neonates and infants: a secondary analysis of the PEPaNIC trial

**DOI:** 10.1007/s00431-023-05298-1

**Published:** 2023-11-11

**Authors:** E. I. Dijkhuizen, K. Dulfer, S. de Munck, N. E. M. van Haren, R. C. J. de Jonge, I. Vanhorebeek, P. J. Wouters, G. Van den Berghe, S. C. A. T. Verbruggen, K. F. M. Joosten

**Affiliations:** 1 Department of Neonatal & Pediatric Intensive Care, Erasmus Medical Center, Sophia Children’s Hospital, Rotterdam, The Netherlands; 2 Department of Child and Adolescent Psychiatry/Psychology, Erasmus Medical Center, Sophia Children’s Hospital, Rotterdam, The Netherlands; 3 Clinical Division and Laboratory of Intensive Care Medicine, Department of Cellular and Molecular Medicine, KU Leuven, Leuven, Belgium

**Keywords:** Pediatric intensive care unit, Neonates, Infants, Neuropsychology, Anthropometrics

## Abstract

Neonates and infants surviving critical illness show impaired growth during critical illness and are at risk for later neuropsychological impairments. Early identification of individuals most at risk is needed to provide tailored long-term follow-up and care. The research question is whether early growth during hospitalization is associated with growth and neuropsychological outcomes in neonates and infants after pediatric intensive care unit admission (PICU). This is a secondary analysis of the PEPaNIC trial. Weight measurements upon PICU admission, at PICU discharge, at hospital discharge, at 2- and 4-year follow-up, and of different subgroups were compared using (paired) *t*-tests. Multiple linear regression analyses were performed to investigate the association between early growth in weight measures and neuropsychological outcomes at 4-year follow-up. One hundred twenty-one infants were included, and median age upon admission was 21 days. Growth in weight per week was less than the age-appropriate norm, resulting in a decrease in weight-for-age *Z*-score during hospitalization. Weight is normalized at 2- and 4-year follow-up. Weight gain in kilograms per week and change in weight *Z*-score were not associated with neurodevelopmental outcome measures at 4-year follow-up. Lower weight-for-age *Z*-score at PICU admission and at hospital discharge was associated only with lower weight and height *Z*-scores at 4-year follow-up.

*Conclusion*: Growth in weight during hospital stay of young survivors of critical illness is impaired. Worse early growth in weight is associated with lower weight and height but not with neuropsychological outcomes at 4-year follow-up.

**Table:** 

**What is Known:** *• Critically ill neonates and infants show impaired early growth during admission and are at risk for later neuropsychological impairments.* *• Unraveling the association between early growth and later neuropsychological impairments is crucial since the first year of life is critical for brain development.*	
**What is New:** *• Critically ill neonates and infants had age appropriate weight measures at 4-year follow-up.* *• Poor growth in weight during hospital stay was not associated with poorer cognitive, emotional, or behavioral functioning four years after critical illness.*

**Supplementary Information:**

The online version contains supplementary material available at 10.1007/s00431-023-05298-1.

## Introduction

The increasing survival rates following pediatric critical illness have led to a shift in focus towards the long-term outcomes of critically ill children [1, 2]. Unfortunately, many survivors are at risk of experiencing later neurocognitive, emotional, or behavioral impairments [3–12]. These neuropsychological impairments have a multifactorial cause, which complicates the accurate risk prediction during and after critical illness [3, 4]. Currently, identifying at-risk patients relies on neuropsychological assessment, but higher-order cognitive functions, such as memory and executive functions, can only be reliably assessed from school-age onwards. As a result, recognizing at-risk patients for academic difficulties often occurs after the manifestation of neuropsychological deficits [13–16]. Implementing preventive interventions earlier in life could positively impact neuropsychological development, leading to a growing interest in identifying early predictors measurable during infancy.

Growth and nutritional outcomes could possibly serve as early predictors, considering that critical illness negatively affects these factors in children during hospitalization and in the post-hospitalization period, with neonates and infants being particularly vulnerable [17–22]. In neonates and infants, poor growth and inadequate nutritional status at the time of admission to the pediatric intensive care unit (PICU) have been linked to unfavorable short-term health outcomes, including prolonged length of PICU stay, increased risk of infection, and increased mortality [23, 24]. However, the association between growth and nutritional status and long-term neuropsychological outcomes, such as neurocognitive functions or emotional and behavioral problems, remains unknown. Unraveling this association in critically ill neonates and infants is crucial since the first year of life is critical for brain development [25–28]. During this time period, the brain is particularly vulnerable for external factors that can influence its development, such as hypoxia, stress, and exposure to anesthetics [15, 29–31]. Given the increased risk of weight deterioration and poor nutritional status in neonates and infants surviving critical illness during this critical time window, further investigation is needed to understand the possible association between growth measures during initial hospital admission and neuropsychological outcomes later in life in this population.

The current study is a secondary analysis of the multicenter, randomized controlled trial (RCT): the pediatric early versus late parenteral nutrition in critical illness (PEPaNIC) trial [32].

This RCT demonstrated that withholding from supplementary parenteral nutrition (PN) during the initial week of admission to the pediatric intensive care unit (PICU) yielded superior short-term outcomes. These benefits encompassed a reduced occurrence of new infections, a shorter duration of stay in the PICU, and diminished direct healthcare expenditures, as opposed to the administration of parenteral nutrition on the day of PICU admission [33]. In the long term, withholding PN for 1 week did not exert adverse effects on survival, anthropometric measurements, health status, or neurocognitive development [7, 8]. Furthermore, it was observed that, at 2 years and 4 years follow-up, children in the delayed-PN group had better scores on parent-reported executive functioning and/or emotional and behavioral problems and/or improved visual-motor integration, in comparison to the children in the early-PN group. Critically ill children aged 29 days to 11 months at time of exposure were identified as most vulnerable to developmental harm evoked by early-PN [34]. Therefore, in the current secondary analysis, we first focus on investigating the weight trajectories of critically ill neonates and infants admitted to the PICU from admission to 4-year follow-up. Second, we aim to identify possible predictors of these weight trajectories. Lastly, we examine the association between weight growth during hospital stay and growth and neuropsychological outcomes at 4-years follow-up.

## Materials and methods

### Study design

This is a secondary analysis of the multicentre pediatric early versus late parenteral nutrition in critical illness (PEPaNIC) trial randomized controlled trial in a subgroup of children aged < 1 year at randomization. The PEPaNIC trial was conducted at the University Hospitals Leuven, Belgium; Erasmus MC – Sophia Children’s Hospital, Rotterdam, the Netherlands; and Stollery Children’s Hospital, Edmonton, AB, Canada. The study included 1440 critically ill infants and children admitted to the pediatric intensive care units of the participating centers between 2012 and 2015. For additional information regarding the trial protocol, see Appendix S1 (Supplementary files). The full study protocol has been published [32]. Additional to the inclusion criteria for the PEPaNIC RCT, inclusion criteria for the current analysis were (1) the availability of anthropometric measurements on admission and on the last day in hospital and (2) the availability of neuropsychological outcomes at 4-year follow-up. Individuals with a syndrome diagnosis (for the applied definition of a syndrome, see Appendix S2 (Supplementary files)) or a non-physiological growth per week more than two times the age-appropriate norm [35, 36] were excluded. As anthropometry at PICU and hospital discharge were only measured in the Erasmus MC-Sophia Children’s Hospital, only children included at this site were eligible for inclusion in this secondary analysis. This study was performed in line with the principles of the Declaration of Helsinki. Approval was granted by the Ethics Committee of Erasmus MC (NL49708.078). Written informed consent was obtained from the parents.

### Weight trajectories from PICU admission to 4-year follow-up

Weight was measured to the nearest 0.01 kg using calibrated scales [24], upon PICU admission, at PICU discharge, at hospital discharge, and at 2- and 4-year follow-up. Early growth was defined as change in weight *Z*-score from admission to the last day in PICU and from admission to the last day in hospital. Weight *Z*-score was defined as weight-for-age *Z*-score in children < 1 year old and BMI-for-age *Z*-score in children ≥ 1 year old [37]. For neonates, birthweight-for-gestational age *Z*-scores [38] were used until the age of seven days, to account for physiological weight loss during the first week of life. Change in weight *Z*-score was categorized as decline (change in weight *Z*-score < 0, rounded to two decimals), no change (change in weight *Z*-score = 0), and incline (change in weight *Z*-score > 0).

To investigate whether clinical parameters predict growth in weight outcomes, weight trajectories were plotted for different subgroups: diagnostic group (surgical cardiac, surgical other, and medical), neonates (age < 1 month) at admission yes/no, malnourished at admission (weight-for-age *Z*-score < −2) yes/no, and malnourished at hospital discharge yes/no.

### 4-year follow-up of neuropsychological outcomes

Outcome was assessed by pediatric psychologists, who were not involved in the management of the patients during their stay in the pediatric intensive care unit. They were strictly masked to treatment allocation. Parents and caregivers were not masked to treatment allocation and were not actively informed about the initial PEPaNIC study results or the 2-year outcome results [7].

For all participants determined as being able to undergo neurocognitive testing (Appendix S3 (Supplementary files)), performance for a broad range of neurocognitive functions and emotional and behavioral problems was scored using age-appropriate, validated, internationally recognized parent-reported questionnaires, and clinical tests with adequate normative data [7]. At the follow-up visit, body weight and height were measured. A clinical neurological examination was done to assess gross neurological abnormalities, and findings were used to determine cognitive testability. Testability was determined by screening the medical file or on clinical judgment before the start of the cognitive assessment by the physician or psychologist and confirmed by the parents or caregivers.

The test battery at the 4-year follow-up assessment included parent-reported questionnaires, i.e., the Behavior Rating Inventory of Executive Function [39] (BRIEF, for executive functioning, expressed in *T* scores, with mean 50 and standard deviation (SD) 10) and the Child Behavior Checklist [40] (CBCL, for emotional and behavioral problems, expressed in *T* scores, with mean 50 and SD 10). On both questionnaires, higher scores indicate more problems.

Additionally, clinical tests consisted of the age-appropriate version of the Wechsler Intelligence Quotient Scale [41, 42] (WPPSI-III-NL, expressed in standard scores, with mean 100 and SD 15), the Beery Developmental Test of Visual-Motor Integration [43] (VMI, expressed in a scaled score, with mean 10 and SD 3), and tasks of the Amsterdam Neuropsychological Task Battery (ANT) [44] (ANT-Baseline Speed (alertness and reaction time expressed in z-scores with mean 0 and SD 1) and ANT-Tapping (motor coordination as number of taps)). For the clinical tests, a higher score indicated better functioning, with the exception of ANT-Baseline Speed.

### Statistical analysis

#### Growth in weight parameters

In case of multiple recorded weight measurements at the same day, the first measurement was used for all measurements registered at that day. Growth per week during hospital stay was calculated by subtracting weight in kg at admission from weight in kg at hospital discharge and dividing the difference by the length of hospital stay in weeks. Plausibility of calculated growth in weight rates was checked, and all individuals with a calculated growth per week of more than two times the age-appropriate average growth per week [35, 36] were excluded.

#### Descriptive statistics

Variables are reported as proportions, mean (SD), or median (interquartile range (IQR)) as appropriate. Descriptive statistics were compared between (sub)groups using (paired) two-sample *t*-tests, Kruskal–Wallis tests, chi-squared test, or ANOVA analysis of variance.

#### Correlation and association analyses

Correlation between growth from hospital admission to 4-year follow-up and outcome measures at 4-year follow-up were assessed by computing a correlation matrix of the univariate association between all complete pairwise observations, using Spearman’s rho.

Multivariable linear regression analyses were done on the pooled imputed dataset (see Appendix S3 (Supplementary files)) with the β-estimates reported to investigate the association between growth in weight *z*-scores during hospitalization and outcome. All multivariable analyses were adjusted for predefined covariates, being sex, diagnostic group (cardiac surgery, other surgery, and medical), age group at admission (neonate versus non-neonate), severity of illness (PIM3 score), parental smoking behavior before admission to the PICU (in-house smoking or no in-house smoking), and occupational level of the parents or caregivers (see Appendix S5 (Supplementary files)). Analyses were not corrected for the treatment strategy, as previous analyses showed no association between treatment strategy and growth during hospital stay [24].

#### Sensitivity analyses

Sensitivity analyses were done by repeating linear regression analyses with the change in growth per week in kilograms instead of weight *Z*-score during hospital stay, as the weight *Z*-score takes into account sex- and age-specific growth norms. Finally, analyses were also done with both weight for (gestational) age *Z*-scores at PICU admission as well as at hospital discharge, to assess the interplay between static anthropometric measurements at the beginning and end of a period of critical illness and neurodevelopmental outcome.

#### Statistical software

*Z*-scores were calculated with use of Growth Analyzer Research Calculation Tool version 4 and Fenton 2013 Preterm Growth Chart version 6. Statistical analyses were done using RStudio (version 4.1.2) [37, 45]. Two-sided *p* values of 0.05 or less were considered statistically significant and were corrected for multiple comparisons by controlling the false discovery rate (FDR).

## Results

**Fig. 1:**
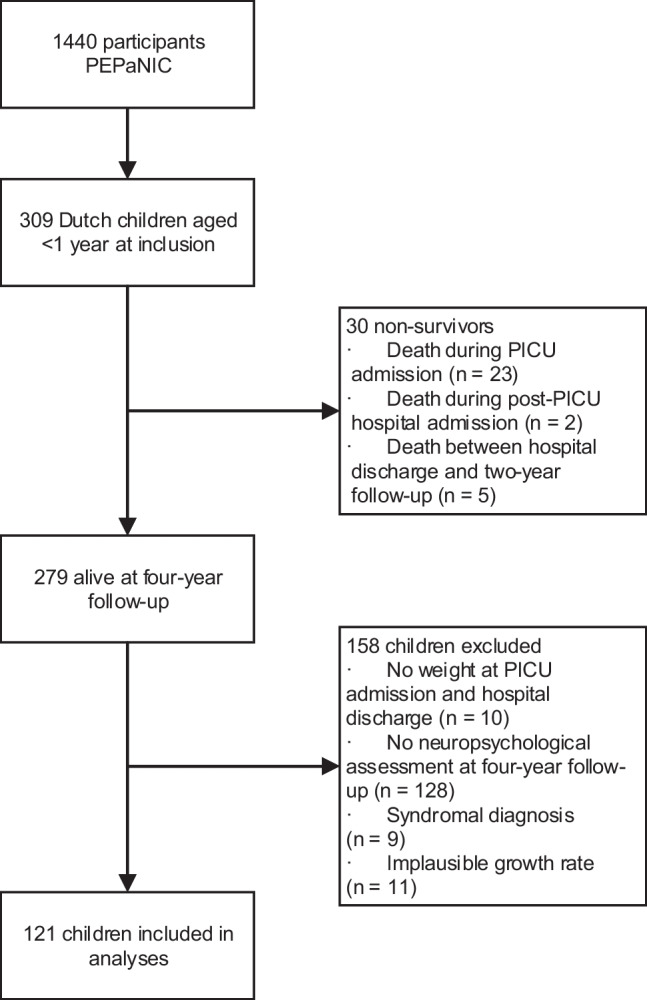
Flowchart of study participants

Of the 309 Dutch PEPaNIC participants aged < 1 year, 121 children were eligible to be included in the analyses; see Fig. 1 for the participants flowchart.

### Baseline characteristics

Baseline characteristics are shown in Table 1. Characteristics of individuals included and not included in the analyses can be found in Table S1 (Supplementary files). Those not included only differed from those included in terms of median duration of hospital stay (included: 13 (8–29) versus not included: 12 (6–24), *p* = 0.048). Patients were evenly distributed among the randomization strategies. Median age at admission was 21 days, 53.7% were neonates, and median age (IQR) at 4-year follow-up was 4.37 (4.22–4.52) years. More detailed information regarding the diagnostic categories of the participants can be found in Table S2 (Supplementary files).

**Table 1:** Demographic and clinical characteristics of the study population

	Overall (*N* = 121)
Demographic and clinical characteristics at PICU admission	
** Randomization strategy (*N* (%))**	
Early PN	59 (48.8%)
Late PN	62 (51.2%)
** Sex (*N* (%))**	
Male	67 (55.4%)
** Age at admission in days (median (IQR))**	21.00 (1.00, 91.00)
** Neonate (age ≤ 28 days) at admission (*N* (%))**	65 (53.7%)
** Age ≤ 7 days at admission in days (N (%))**	47 (38.8%)
** PeLOD score**^a^ **(median (IQR))**	12.0 (3.0, 12.0)
** PIM3 score**^b^ **(median (IQR))**	−3.7 (−4.4, −2.4)
** Elective or urgent admission (*N* (%))**	
Elective	30 (24.8%)
Urgent	91 (75.2%)
** Diagnostic group; cardiac surgery, other surgery versus medical (*N* (%))**	
Surgical—cardiac	27 (22.3%)
Surgical—other	49 (40.5%)
Medical	45 (37.2%)
** STRONGkids risk category**^c^ **(*N* (%))**	
High risk	34 (28.1%)
Medium risk	87 (71.9%)
Demographic characteristics at 4-year follow-up	
** Known non-European origin**^d^ **(*N* (%))**	26 (21.7%)
** Known non-Caucasian origin**^d^ **(*N* (%))**	16 (13.3%)
** Exclusive Dutch or English spoken upbringing (*N* (%))**	95 (79.2%)
** Education level of parents**^e^ **(*N* (%))**	
1 or 1.5	12 (10.0%)
2	25 (20.8%)
2.5	25 (20.8%)
3	32 (26.7%)
Unknown	26 (21.7%)
** Occupation level of parents**^f^ **(*N* (%))**	
1.5 or 2	22 (18.3%)
2.5 or 3	31 (25.8%)
3.5 or 4	31 (25.8%)
Unknown	36 (30.0%)
** In-house smoking between birth and PICU admission (*N* (%))**	43 (35.8%)

*PICU* pediatric intensive care unit, *PN* parenteral nutrition, *PeLOD* pediatric logistic organ dysfunction score, *PIM3* pediatric index of mortality 3 score, *STRONGkids* Screening Tool for Risk on Nutritional Status and Growth

^a^PeLOD scores range from 0 to 71, with higher scores indicating more severe illness

^b^Higher PIM3 scores indicate a higher risk of mortality

^c^STRONGkids scores range from 0 to 5, with a score of 0 indicating a low risk of malnutrition, a score of 1 to 3 indicating a medium risk, and a score of 4 to 5 indicating a high risk

^d^Participants were classified according to race and geographical origin by the investigators

^e^The average of the paternal and maternal educational level and calculated based upon the 3-point scale subdivisions as made by the Centraal Bureau voor de Statistiek. For further definition and calculation, see Appendix S4 (Supplementary files)

^f^The average of the paternal and maternal occupation level is calculated based upon the International Isco System 4-point scale for professions. For further definition and calculation, see Appendix S4 (Supplementary files)

### Early growth in weight parameters

The mean weight *Z*-scores of critically ill neonates and infants during PICU admission, at PICU discharge, and at hospital discharge differed significantly from age- and sex-specific population means, see Table S3 (Supplementary files). At 4-year follow-up, weight *Z*-scores did not differ from age and sex-specific population means. Twenty-four percent of the infants had acute malnutrition (weight for age *Z*-score ≤ −2) on admission; at PICU and hospital discharge, this was 25.6% and 28.1%, respectively. During follow-up, the percentage of malnutrition decreased to 3.8% at 2-year follow-up and 8.3% at 4-year follow-up.

**Fig. 2:**
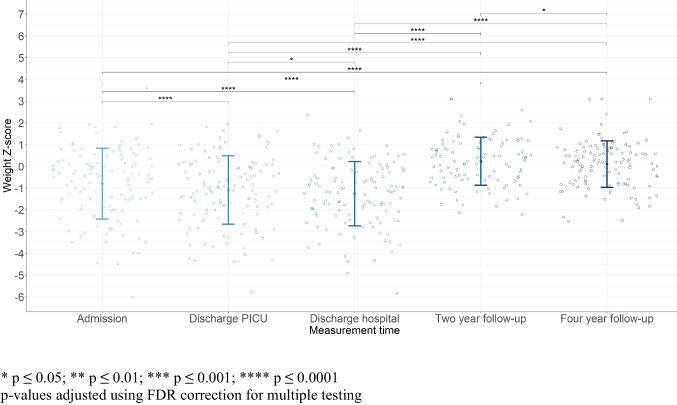
Mean and standard deviation weight *Z*-scores

Mean weight *Z*-scores declined during hospital stay (−0.46 (0.82), *p* < 0.0001). The decline in *Z*-score was greatest in the PICU (−0.29 (0.64), *p* < 0.0001); the mean *Z*-score also declined from discharge from the PICU up to discharge from the hospital (−0.17 (0.72), *p* = 0.012) (Fig. 2). Median weight gain per week in kilograms was mostly below age-appropriate norms for the different age groups and phases of hospital admission (Table S4 (Supplementary files)).

Follow-up measurements at 2- and 4-year post-PICU admission showed a significant increase in weight *Z*-score compared to all measurements during hospital admission. Weight *Z*-score decreased from 2- to 4-year follow-up (−0.20 (0.94), *p* = 0.032) (Table S5 (Supplementary files).

### Predictors for change in weight Z-score from PICU admission to follow-up

**Fig. 3:**
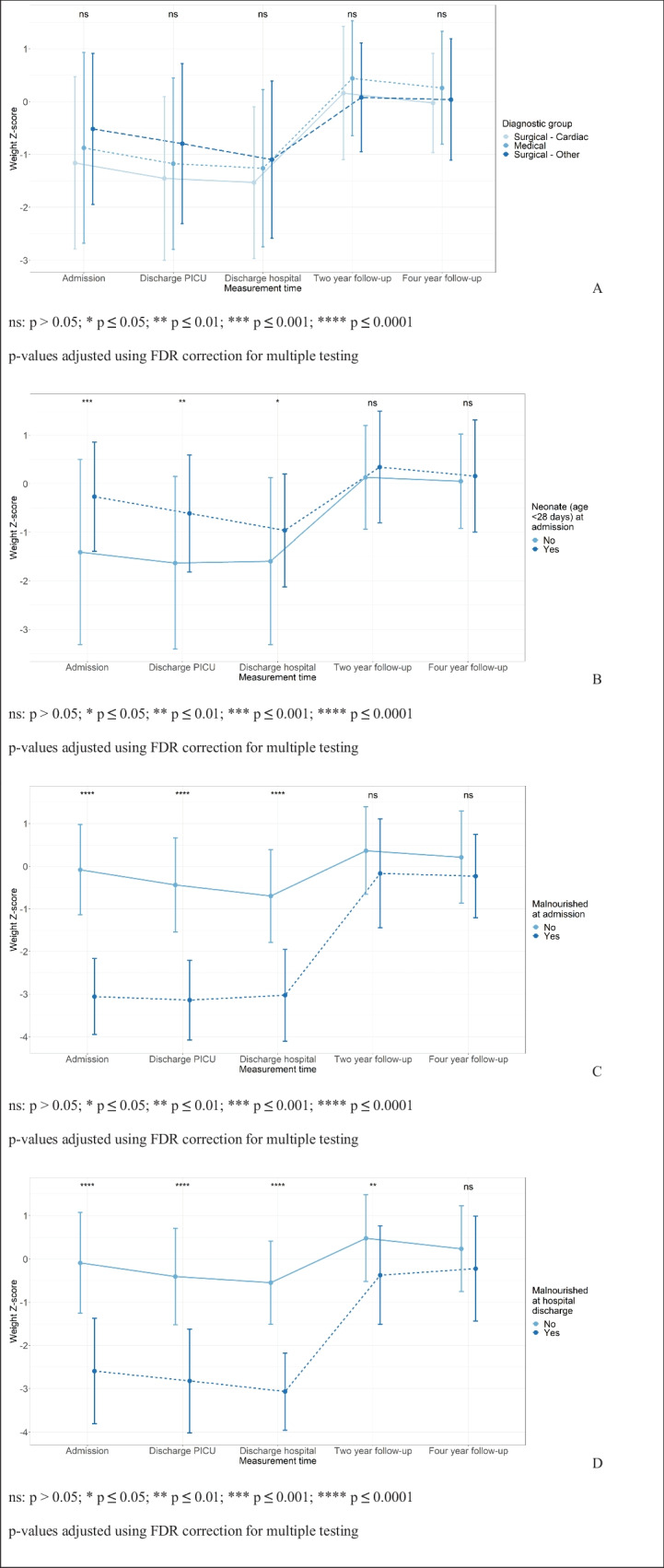
Weight *Z*-score trajectories in different subgroups. **A** Weight Z-score trajectory per diagnostic group, **B** Weight Z-score trajectory neonates versus non-neonates, **C** Weight Z-score trajectory by nutritional status at admission, **D** Weight Z-score trajectory by nutritional status at hospital discharge

Diagnosis category upon admission was not significantly associated with weight *Z*-scores during hospital admission nor at follow-up (Fig. 3A and Table S6 (Supplementary files)). Neonates were admitted and discharged from PICU and hospital with a higher mean weight *Z*-score than non-neonates (Fig. 3B and Table S6 (Supplementary files)). Patients diagnosed with malnutrition (weight for age *Z*-score ≤ −2) at PICU admission also had a lower weight for age *Z*-score during hospitalization than those with no malnutrition at PICU admission (Fig. 3C and Table S6 (Supplementary files)). Those diagnosed with malnutrition at hospital discharge already had a lower mean weight for age *Z*-score at PICU admission compared to patients with no malnutrition at hospital discharge. Mean weight *Z*-score increased from hospital discharge to 2-year follow-up in both groups (mean increase of 2.67 (1.38), *p* < 0.0001 and 1.03 (1.25), *p* < 0.0001). At 2-year follow-up, patients with malnutrition at hospital discharge still had a lower mean weight *Z*-score compared to those without malnutrition at hospital discharge (Fig. 3D and Table S6 (Supplementary files)). This difference had disappeared by 4-year follow-up.

### Associations between early growth during hospitalization and anthropometric and neuropsychological outcomes at 4-year follow-up

Change in weight *Z*-score and change in weight gain in kilograms per week during hospitalization were not significantly associated with growth and neuropsychological outcome at 4-year follow-up (Tables 2 and S7). Weight for (gestational) age *Z*-score at PICU admission was associated with height (β 0.22, 95%CI 0.10–0.35, *p* = 0.014) and weight *Z*-scores (β 0.20, 95%CI 0.08–0.33, *p* = 0.022) at 4-year follow-up (Table S8). Weight for (gestational) age *Z*-score at hospital discharge was associated with weight *Z*-score at 4-year follow-up (β 0.24, 95%CI 0.11–0.38, *p* = 0.014) (Table S9). Anthropometric and neuropsychological outcomes assessed at 4-year follow-up were not correlated (Fig. S2).

**Table 2:** Associations between change in weight *Z*-score during hospital admission and anthropometrics and neuropsychological outcomes at 4-year follow-up

	**Number (%) of observations**	**Pooled outcome**	**Univariable analysis**		**Multivariable analysis**^a^	
	Total (*N* = 121)	Mean (SD)	β-Estimate (95%CI)	Adjusted *p* value	β-Estimate (95%CI)	Adjusted *p* value
**Anthropometrics**						
Height *Z*-score^b^	121 (100%)	0.16 (1.07)	−0.30 (−0.53, −0.07)	0.275	−0.26 (−0.51, −0.018)	0.557
Weight *Z*-score^b^	121 (100%)	0.11 (1.07)	−0.04 (−0.27, 0.20)	0.976	0.01 (−0.24, 0.26)	0.989
BMI *Z*-score^b^	120 (99.2%)	0.03 (1.20)	0.20 (−0.07, 0.46)	0.976	0.25 (−0.02, 0.52)	0.582
**Executive functions (BRIEF**^c**)**^						
Emotional control	121 (100%)	50.1 (10.5)	0.01 (−2.33, 2.35)	0.995	−0.17 (−2.73, 2.39)	0.989
Flexibility	121 (100%)	50.5 (11.6)	−1.00 (−3.58, 1.58)	0.976	−1.37 (−4.19, 1.45)	0.847
Inhibition	121 (100%)	50.5 (11.5)	0.41 (−2.16, 2.97)	0.976	0.35 (− 2.43, 3.13)	0.956
Working memory	121 (100%)	51.7 (11.9)	0.26 (−2.37, 2.90)	0.976	0.42 (−2.44, 3.28)	0.956
Meta-cognition index	121 (100%)	51.0 (11.3)	0.41 (−2.10, 2.92)	0.976	0.61 (−2.12, 3.34)	0.917
Planning and organization	121 (100%)	50.4 (10.2)	0.63 (−1.64, 2.89)	0.976	0.82 (−1.66, 3.30)	0.847
Total score	121 (100%)	50.7 (11.9)	0.08 (−2.57, 2.73)	0.993	0.06 (−2.83, 2.94)	0.989
**Emotional and behavioral problems (CBCL**^c^**)**						
Externalizing problems	121 (100%)	47.3 (10.2)	0.42 (−1.84, 2.69)	0.976	0.75 (−1.60, 3.09)	0.847
Internalizing problems	121 (100%)	49.4 (10.6)	−1.16 (−3.51, 1.19)	0.976	−0.91 (−3.39, 1.57)	0.847
Total	121 (100%)	48.0 (10.7)	−0.47 (−2.85, 1.92)	0.976	−0.02 (−2.48, 2.44)	0.989
**IQ**^d^						
Performal IQ	121 (100%)	92.3 (13.1)	1.54 (−1.36, 4.44)	0.976	1.83 (−1.22, 4.88)	0.847
Verbal IQ	121 (100%)	96.6 (16.8)	0.38 (−3.35, 4.11)	0.976	1.22 (−2.67, 5.11)	0.847
Total IQ	121 (100%)	92.7 (15.0)	1.03 (−2.29, 4.35)	0.976	1.74 (−1.71, 5.18)	0.847
**Motor coordination**^d^						
Number of unimanual taps (left hand)	101 (83.5%)	23.4 (6.1)	−0.39 (−1.86, 1.07)	0.976	−0.49 (−2.13, 1.14)	0.847
Number of unimanual taps (right hand)	101 (83.5%)	25.7 (6.6)	0.60 (−0.99, 2.18)	0.976	0.50 (−1.15, 2.15)	0.847
Number of valid alternating taps	101 (83.5%)	11.8 (8.6)	−2.03 (−4.08, 0.02)	0.647	−2.28 (− 4.51, −0.056)	0.557
Number of valid synchronous taps	101 (83.5%)	7.5 (6.0)	−0.99 (−2.44, 0.45)	0.976	−1.31 (−2.91, 0.29)	0.667
**Alertness**^c^						
Reaction time left hand (*Z*-score)	101 (83.5%)	1.6 (1.4)	0.09 (−0.25, 0.43)	0.976	0.14 (−0.24, 0.52)	0.847
Within subject SD of repeated tests (*Z*-score)	101 (83.5%)	2.3 (1.2)	0.04 (−0.26, 0.34)	0.976	0.11 (−0.22, 0.45)	0.847
Reaction time right hand (*Z*-score)	101 (83.5%)	2.6 (1.5)	−0.02 (−0.39, 0.35)	0.976	0.06 (−0.35, 0.47)	0.956
Within subject SD of repeated tests (*Z*-score)	101 (83.5%)	3.0 (1.4)	−0.03 (−0.38, 0.32)	0.976	0.12 (−0.27, 0.50)	0.847
**Visual motor-integration**^d^	121 (100%)	9.8 (2.2)	0.10 (−0.40, 0.59)	0.976	0.15 (−0.37, 0.66)	0.847

Results are presented in numbers with proportions (%), mean (SD), and β-estimates (95%CI)

*BMI* body mass index, *BRIEF* Behavior Rating Inventory of Executive Function (parent-reported), *CBCL* Child Behavior Checklist (parent-reported), *IQ* intelligence quotient, *SES* socio-economic status

^a^Adjusted for sex, diagnostic group, age group at admission, a predefined syndrome, PIM3 score, parental smoking behavior before admission to the PICU, and occupational level of the parents or caregivers

^b^Age-sex-adjusted *Z*-scores were calculated with the use of reference data from Fenton- and nationally available growth charts

^c^Higher scores reflect worse performance

^d^Higher scores reflect better performance. *p* values adjusted using FDR correction for multiple testing

## Discussion

In this secondary analysis of the PEPaNIC randomized control trial, we found that both change in weight-for-age *Z*-score and growth in kilograms per week were not associated with anthropometric or neuropsychological outcomes at 4-year follow-up. As to their weight trajectories during hospitalization, weight gain was markedly less than age- and sex-appropriate norms, expressed in a decrease in weight *Z*-score and a mean growth per week in kilograms below the age-specific norms. Moreover, we found a high, persistent prevalence of malnutrition during hospitalization, with the proportion of neonates and infants fulfilling the criteria for acute malnutrition (weight-for-age *Z*-score < −2) increasing from 24.0% at PICU admission, to 25.6% at PICU discharge, and 28.1% at hospital discharge.

This high prevalence of malnutrition is in accordance with previous studies in which a prevalence of malnutrition op to up to 24% has been found [46, 47]. However, in the years following critical illness, critically ill neonates and infants appear to be able to catch up, also those who were malnourished upon admission. In this study, most participants with malnutrition on PICU admission were also malnourished at hospital discharge (22 out of 27, 81.5%). As to what might explain this, LOS can be of interest. We did not investigate a possible association between LOS and change in nutritional status during hospitalization. However, no difference in LOS between those who were malnourished on PICU admission and those who were not, nor between those who were malnourished at hospital discharge and those who were not. With a median LOS of 13 days, this should be long enough to expect sufficient weight gain in most study participants. Regarding the difference in weight *Z*-score between those who were malnourished at hospital discharge and those who were not persevering to 2-year follow-up, but disappearing at 4-year follow-up, this may indicate the amount of time needed for these young children to overcome the disadvantage they started off with due to their critical illness.

The association between anthropometric measures and short term, medical outcome has been studied frequently. Poor nutritional status had been associated with longer duration of mechanical ventilation [48–51], longer duration of PICU stay [23, 47, 49, 51–53], and mortality [23, 51–54]. In a cohort of children with congenital heart disease, impaired weight gain was predictive of late post-surgical mortality [19]. A secondary analysis of the PEPaNIC RCT has shown that less decrease in weight *Z*-score during PICU admission was associated with a lower risk of new infection and a higher likelihood of earlier alive discharge from PICU [24].

On the other hand, the association between weight gain or other anthropometric measures during hospital stay and long-term, neuropsychological outcome has, to our knowledge, only been studied in children undergoing surgery for congenital heart disease [55–58]. In a study of 107 infants undergoing open-heart surgery for congenital heart disease, neurodevelopmental outcome at one year was not associated with growth failure. It was shown that impaired weight gain before surgery was followed by catch-up growth after surgery [57]. Also, in another study of 143 infants, impaired intellectual abilities at 6 years were not associated with growth in weight [58]. Only in a study of 72 infants with congenital heart disease 3-month weight-for-age *Z*-score was associated with lower mental developmental index and psychomotor developmental index scores at 12 months [56].

Within the PEPaNIC RCT that included the total PICU population, it has been demonstrated that withholding supplementary parenteral nutrition during the first week of a PICU admission did not harm physical and neurocognitive development assessed 2 or 4 years after critical illness, compared to early-parenteral nutrition [7, 8]. So, even though macronutrient intake was less in the late PN-group, there was no effect of PN strategy on weight *Z*-trajectories [24, 33]. Moreover, analyses evaluating the role of age at PICU admission on the effect of early PN showed that children aged 29 days to 11 months were most vulnerable for the observed developmental harm of early PN [34]. These previous PEPaNIC findings together with the lack of association between growth during hospital stay and 4-year neuropsychological outcomes of our current study suggest that inability to achieve intake targets and even subsequent impaired growth in weight during critical illness, especially in infants, may not be detrimental for longer term outcome of PICU survivors, opposed to what has been proposed before [55, 56, 59, 60].

Question remains what factors are associated with the observed worse outcome of young PICU survivors in some neuropsychological domains. In previous studies in infants with congenital heart disease, impaired height trajectories were associated with worse neurodevelopmental outcomes [55, 56]. However, head circumference at birth, postnatal factors, genetic comorbidity, and epigenetic changes are important factors which must be taken into account concerning impaired height trajectories and neuropsychological outcome [61]. Also, when evaluating outcome of children after PICU discharge, it is important to include baseline (health) status and family factors in multifactorial predictive models [62, 63]. The use of such predictive models to identify those critically ill children most at risk already during PICU stay is clinically relevant, as it enables the possibility for tailored long-term follow-up, which is essential to prevent further deviation of normal development as much as possible. Relevance of early identification of predictive factors is further underlined by an analysis of the change in neurodevelopmental outcome of PEPaNIC participants from 2- to 4-year post-PICU admission [64]. This analysis showed that developmental impairments remained prominent in the general PICU population group. Within the investigated time-window, impaired growth in height, impairment in executive functioning, and in intelligence aggravated. Based on these findings, it was determined that additional investigation is necessary to understand the long-term impact of pediatric critical illness on development into adulthood [64].

### Limitations

The major strength of this study is the longitudinal growth assessment of a large group of former PICU neonates and infants and their neuropsychological functioning at 4-year follow-up.

The study has several limitations despite its strengths. First, since only a subset of the total trial population was analyzed, there may be selection bias. However, this bias is unlikely as participation was based mainly on the center rather than clinical variables that could influence the association being investigated. Additionally, a comparison of baseline and demographic characteristics between infants from Rotterdam included and not included in this showed no differences. As this analysis composes of only a subgroup of the PEPaNIC trial, not all covariates corrected for in the original PEPaNIC analyses [7, 8] could be added in the models of this analysis. Therefore, the overall effect of the association of weight gain and neuropsychological outcome may be diminished.

Second, the study relied on weight measurements to evaluate growth, which may be affected by factors like edema and fluid retention during illness. To mitigate this issue, growth parameters were examined throughout the entire hospital admission, not just during PICU stay. The weight measurements followed a consistent protocol for all participants, reducing the likelihood of unreliable, and potentially biased measurements. Unfortunately, assessing body composition through methods like air plethysmography, dual X-ray absorptiometry, or bioelectrical impedance was not feasible for critically ill children. Alternatively, mid-upper arm or calf circumference could have been used to estimate body composition, but the available measurements in the cohort were insufficient for regression analysis. Head circumference, another relevant anthropometric measure for neurodevelopment assessment [58, 65], was not recorded during the hospital stay for critical illness in this cohort.

Lastly, the use of the pooled imputed dataset for drawing inferences may underestimate the errors around the model coefficients. However, the obtained estimates remain unbiased.

### Conclusion

Weight gain during hospital stay of critically ill neonates and infants is insufficient, resulting in growth in kilograms per week less than age- and sex-appropriate norms and thus a decrease in weight-for-age *Z*-score throughout hospital stay.

Although neuropsychological outcome is impaired in neonates and infants after critical illness, poor growth in weight during hospital stay was not associated with poorer cognitive, emotional, or behavioral functioning 4 years after critical illness.

Further research should focus on the effects of impaired growth during infancy on development into adulthood, extending beyond weight as growth parameter and looking into other outcome measures, assessed at a later age, as well. Moreover, additional research should try to identify early factors predictive of poorer neurocognitive functioning after critical illness in neonates and infants.

### Supplementary Information

Below is the link to the electronic supplementary material.

Supplementary file1 (DOCX 480 KB)

## Data Availability

Data sharing will be considered only on a collaborative basis with the principal investigators, after evaluation of the proposed study protocol and statistical analysis plan.

## Abbreviations

FDR: False discovery rate
PeLOD: Pediatric logistic organ dysfunction score
PEPaNIC: The early versus late parenteral nutrition in the pediatric intensive care unit trial
PICU: Pediatric intensive care unit
PIM3: Pediatric index of mortality 3
PN: Parenteral nutrition
STRONGkids: Screening Tool for Risk on Nutritional Status and Growth

## References

[1] Euro-Peristat Project. European Perinatal Health Report. Core indicators of the health and care of pregnant women and babies in Europe in 2015. November 2018. https://www.europeristat.com/

[2] Broeders L, Achterberg PW, Waelput AJM, Ravelli ACJ, Kwee A, Groenendaal F, Offerhaus P, van der Velden K, Rosman AN, Nijhuis JG (2019) Decrease in foetal and neonatal mortality in the Netherlands; comparison with other Euro-Peristat countries in 2004, 2010 and 2015 Afname van foetale en neonatale sterfte in Nederland. Ned Tijdschr Geneeskd 163 31361412

[3] White BR, Rogers LS, Kirschen MP (2019). Recent advances in our understanding of neurodevelopmental outcomes in congenital heart disease. Curr Opin Pediatr.

[4] Verstraete S, Van den Berghe G, Vanhorebeek I (2018). What’s new in the long-term neurodevelopmental outcome of critically ill children. Intensive Care Med.

[5] Bellinger DC, Wypij D, Rivkin MJ, DeMaso DR, Robertson RL, Dunbar-Masterson C, Rappaport LA, Wernovsky G, Jonas RA, Newburger JW (2011). Adolescents with d-transposition of the great arteries corrected with the arterial switch procedure: neuropsychological assessment and structural brain imaging. Circulation.

[6] Goldberg CS, Lu M, Sleeper LA, Mahle WT, Gaynor JW, Williams IA, Mussatto KA, Ohye RG, Graham EM, Frank DU, Jacobs JP, Krawczeski C, Lambert L, Lewis A, Pemberton VL, Sananes R, Sood E, Wechsler SB, Bellinger DC, Newburger JW, Pediatric Heart Network I (2014). Factors associated with neurodevelopment for children with single ventricle lesions. J Pediatr.

[7] Jacobs A, Dulfer K, Eveleens RD, Hordijk J, Van Cleemput H, Verlinden I, Wouters PJ, Mebis L, Guerra GG, Joosten K, Verbruggen SC, Guiza F, Vanhorebeek I, Van den Berghe G (2020). Long-term developmental effect of withholding parenteral nutrition in paediatric intensive care units: a 4-year follow-up of the PEPaNIC randomised controlled trial. Lancet Child Adolesc Health.

[8] Verstraete S, Verbruggen SC, Hordijk JA, Vanhorebeek I, Dulfer K, Guiza F, van Puffelen E, Jacobs A, Leys S, Durt A, Van Cleemput H, Eveleens RD, Garcia Guerra G, Wouters PJ, Joosten KF, Van den Berghe G (2019). Long-term developmental effects of withholding parenteral nutrition for 1 week in the paediatric intensive care unit: a 2-year follow-up of the PEPaNIC international, randomised, controlled trial. Lancet Respir Med.

[9] Madderom MJ, Reuser JJ, Utens EM, van Rosmalen J, Raets M, Govaert P, Steiner K, Gischler SJ, Tibboel D, van Heijst AF, Ijsselstijn H (2013). Neurodevelopmental, educational and behavioral outcome at 8 years after neonatal ECMO: a nationwide multicenter study. Intensive Care Med.

[10] Mesotten D, Gielen M, Sterken C, Claessens K, Hermans G, Vlasselaers D, Lemiere J, Lagae L, Gewillig M, Eyskens B, Vanhorebeek I, Wouters PJ, Van den Berghe G (2012). Neurocognitive development of children 4 years after critical illness and treatment with tight glucose control: a randomized controlled trial. JAMA.

[11] Newburger JW, Sleeper LA, Bellinger DC, Goldberg CS, Tabbutt S, Lu M, Mussatto KA, Williams IA, Gustafson KE, Mital S, Pike N, Sood E, Mahle WT, Cooper DS, Dunbar-Masterson C, Krawczeski CD, Lewis A, Menon SC, Pemberton VL, Ravishankar C, Atz TW, Ohye RG, Gaynor JW, Pediatric Heart Network I (2012). Early developmental outcome in children with hypoplastic left heart syndrome and related anomalies: the single ventricle reconstruction trial. Circulation.

[12] Hordijk JA, Verbruggen SC, Buysse CM, Utens EM, Joosten KF, Dulfer K (2022). Neurocognitive functioning and health-related quality of life of children after pediatric intensive care admission: a systematic review. Qual Life Res.

[13] Marino BS, Lipkin PH, Newburger JW, Peacock G, Gerdes M, Gaynor JW, Mussatto KA, Uzark K, Goldberg CS, Johnson WH, Li J, Smith SE, Bellinger DC, Mahle WT, American Heart Association Congenital Heart Defects Committee CoCDitYCoCN, Stroke C (2012). Neurodevelopmental outcomes in children with congenital heart disease: evaluation and management: a scientific statement from the American Heart Association. Circulation.

[14] Sterken C, Lemiere J, Vanhorebeek I, Van den Berghe G, Mesotten D (2015). Neurocognition after paediatric heart surgery: a systematic review and meta-analysis. Open Heart.

[15] Schiller R, Ijsselstijn H, Hoskote A, White T, Verhulst F, van Heijst A, Tibboel D (2018) Memory deficits following neonatal critical illness: a common neurodevelopmental pathway. Lancet Child Adolesc Health 2:281–289 10.1016/S2352-4642(17)30180-3 30169299

[16] Anderson PJ (2014). Neuropsychological outcomes of children born very preterm. Semin Fetal Neonatal Med.

[17] Hulst J, Joosten K, Zimmermann L, Hop W, van Buuren S, Buller H, Tibboel D, van Goudoever J (2004). Malnutrition in critically ill children: from admission to 6 months after discharge. Clin Nutr.

[18] Bairdain S, Khan FA, Fisher J, Zurakowski D, Ariagno K, Cauley RP, Zalieckas J, Wilson JM, Jaksic T, Mehta NM (2015). Nutritional outcomes in survivors of congenital diaphragmatic hernia (CDH)-factors associated with growth at one year. J Pediatr Surg.

[19] Eskedal LT, Hagemo PS, Seem E, Eskild A, Cvancarova M, Seiler S, Thaulow E (2008). Impaired weight gain predicts risk of late death after surgery for congenital heart defects. Arch Dis Child.

[20] Karpen HE (2016). Nutrition in the cardiac newborns: evidence-based nutrition guidelines for cardiac newborns. Clin Perinatol.

[21] Hulst JM, van Goudoever JB, Zimmermann LJ, Hop WC, Albers MJ, Tibboel D, Joosten KF (2004). The effect of cumulative energy and protein deficiency on anthropometric parameters in a pediatric ICU population. Clin Nutr.

[22] Kaufman J, Vichayavilas P, Rannie M, Peyton C, Carpenter E, Hull D, Alpern J, Barrett C, da Cruz EM, Roosevelt G (2015) Improved nutrition delivery and nutrition status in critically ill children with heart disease. Pediatrics 135:e717–725 10.1542/peds.2014-1835 25687139

[23] Ross FJ, Radman M, Jacobs ML, Sassano-Miguel C, Joffe DC, Hill KD, Chiswell K, Feng L, Jacobs JP, Vener DF, Latham GJ (2020). Associations between anthropometric indices and outcomes of congenital heart operations in infants and young children: an analysis of data from the Society of Thoracic Surgeons Database. Am Heart J.

[24] van Puffelen E, Hulst JM, Vanhorebeek I, Dulfer K, Van den Berghe G, Joosten KFM, Verbruggen S (2020). Effect of late versus early initiation of parenteral nutrition on weight deterioration during PICU stay: secondary analysis of the PEPaNIC randomised controlled trial. Clin Nutr.

[25] Bhutta ZA, Guerrant RL, Nelson CA (2017). Neurodevelopment, nutrition, and inflammation: the evolving global child health landscape. Pediatrics.

[26] Fox SE, Levitt P, Nelson CA (2010). How the timing and quality of early experiences influence the development of brain architecture. Child Dev.

[27] Kolb B, Fantie BD (2009) Development of the child’s brain and behavior. Handbook of clinical child neuropsychology. Springer Science + Business Media, New York, pp 19–46

[28] Knickmeyer RC, Gouttard S, Kang C, Evans D, Wilber K, Smith JK, Hamer RM, Lin W, Gerig G, Gilmore JH (2008). A structural MRI study of human brain development from birth to 2 years. J Neurosci.

[29] Claessens NHP, Kelly CJ, Counsell SJ, Benders M (2017). Neuroimaging, cardiovascular physiology, and functional outcomes in infants with congenital heart disease. Dev Med Child Neurol.

[30] Ortinau CM, Shimony JS (2020). The congenital heart disease brain: prenatal considerations for perioperative neurocritical care. Pediatr Neurol.

[31] Peyvandi S, Latal B, Miller SP, McQuillen PS (2019). The neonatal brain in critical congenital heart disease: insights and future directions. Neuroimage.

[32] Fivez T, Kerklaan D, Verbruggen S, Vanhorebeek I, Verstraete S, Tibboel D, Guerra GG, Wouters PJ, Joffe A, Joosten K, Mesotten D, Van den Berghe G (2015). Impact of withholding early parenteral nutrition completing enteral nutrition in pediatric critically ill patients (PEPaNIC trial): study protocol for a randomized controlled trial. Trials.

[33] Fivez T, Kerklaan D, Mesotten D, Verbruggen S, Wouters PJ, Vanhorebeek I, Debaveye Y, Vlasselaers D, Desmet L, Casaer MP, Garcia Guerra G, Hanot J, Joffe A, Tibboel D, Joosten K, Van den Berghe G (2016). Early versus late parenteral nutrition in critically ill children. N Engl J Med.

[34] Verlinden I, Dulfer K, Vanhorebeek I, Guiza F, Hordijk JA, Wouters PJ, Guerra GG, Joosten KF, Verbruggen SC, Van den Berghe G (2021). Role of age of critically ill children at time of exposure to early or late parenteral nutrition in determining the impact hereof on long-term neurocognitive development: a secondary analysis of the PEPaNIC-RCT. Clin Nutr.

[35] Kindergeneeskunde NVV (2021). Ondervoeding bij kinderen.

[36] Dubey AP (2015). Pediatric nutrition in practice.

[37] Talma H (2010) Groeidiagrammen TNO (Dutch Growth Standards). https://www.tno.nl/nl/gezond/werk-jeugd-gezondheid/jeugd/eerste-1000-dagen-kind/groeidiagrammen-groeicalculators/

[38] Fenton TR, Kim JH (2013). A systematic review and meta-analysis to revise the Fenton growth chart for preterm infants. BMC Pediatr.

[39] van der Heijden KB, Suurland J, De Sonneville LM, Swaab HJ (2013) BRIEF-P: Vragenlijst executieve functies voor 2- tot 5-jarigen - Handleiding. Hogrefe, Amsterdam

[40] Achenbach TM, Rescorla LA (2000) Manual for the ASEBA preschool forms and profiles. University of Vermont, Research Center for Children, Youth and Families, Burlington, VT

[41] Hendriksen JG, Hurks PP (2010) WPPSI-III-NL Wechsler preschool and primary scale of intelligence. Nederlandstalige bewerking: handleiding. Pearson, Amsterdam

[42] Hurks PP, Hendriksen JG, Dek J, Kooij A (2010) De nieuwe Wechsler kleuterintelligentietest voor 2:6–7:11 jarigen. Tijdschrift voor Neuropsychologie 2:40–51

[43] Beery KEB, Beery NA (2010) The Beery-Buktenica developmental test of visual-motor integration, 6th edition (BEERY^™^ VMI). Pearson, Amsterdam

[44] De Sonneville LMJ (2005) Amsterdamse Neuropsychologische Taken: Wetenschappelijke en klinische toepassingen [Amsterdam neuropsychological tasks: scientific and clinical applications]. Tijdschrift voor Neuropsychologie 0:27–41

[45] RStudio Team (2015) RStudio: integrated development environment for R [Internet]. Boston, MA

[46] Pollack MM, Wiley JS, Holbrook PR (1981). Early nutritional depletion in critically ill children. Crit Care Med.

[47] Valla FV, Berthiller J, Gaillard-Le-Roux B, Ford-Chessel C, Ginhoux T, Rooze S, Cour-Andlauer F, Meyer R, Javouhey E (2018). Faltering growth in the critically ill child: prevalence, risk factors, and impaired outcome. Eur J Pediatr.

[48] Grippa RB, Silva PS, Barbosa E, Bresolin NL, Mehta NM, Moreno YM (2017). Nutritional status as a predictor of duration of mechanical ventilation in critically ill children. Nutrition.

[49] Bagri NK, Jose B, Shah SK, Bhutia TD, Kabra SK, Lodha R (2015). Impact of malnutrition on the outcome of critically ill children. Indian J Pediatr.

[50] de Souza MF, Leite HP, Koch Nogueira PC (2012). Malnutrition as an independent predictor of clinical outcome in critically ill children. Nutrition.

[51] Ross F, Latham G, Joffe D, Richards M, Geiduschek J, Eisses M, Thompson D, Radman M (2017). Preoperative malnutrition is associated with increased mortality and adverse outcomes after paediatric cardiac surgery. Cardiol Young.

[52] van Puffelen E, Hulst JM, Vanhorebeek I, Dulfer K, Van den Berghe G, Verbruggen S, Joosten KFM (2018). Outcomes of delaying parenteral nutrition for 1 week vs initiation within 24 hours among undernourished children in pediatric intensive care: a subanalysis of the PEPaNIC randomized clinical trial. JAMA Netw Open.

[53] Irving SY, Daly B, Verger J, Typpo KV, Brown AM, Hanlon A, Weiss SL, Fitzgerald JC, Nadkarni VM, Thomas NJ, Srinivasan V, Sepsis Prevalence O, Therapies Study I, Pediatric Acute Lung I, Sepsis Investigators N (2018). The association of nutrition status expressed as body mass index z score with outcomes in children with severe sepsis: a secondary analysis from the Sepsis Prevalence, Outcomes, and Therapies (SPROUT) Study. Crit Care Med.

[54] Prince NJ, Brown KL, Mebrahtu TF, Parslow RC, Peters MJ (2014). Weight-for-age distribution and case-mix adjusted outcomes of 14,307 paediatric intensive care admissions. Intensive Care Med.

[55] Ravishankar C, Zak V, Williams IA, Bellinger DC, Gaynor JW, Ghanayem NS, Krawczeski CD, Licht DJ, Mahony L, Newburger JW, Pemberton VL, Williams RV, Sananes R, Cook AL, Atz T, Khaikin S, Hsu DT, Pediatric Heart Network I (2013). Association of impaired linear growth and worse neurodevelopmental outcome in infants with single ventricle physiology: a report from the pediatric heart network infant single ventricle trial. J Pediatr.

[56] Medoff-Cooper B, Irving SY, Hanlon AL, Golfenshtein N, Radcliffe J, Stallings VA, Marino BS, Ravishankar C (2016). The association among feeding mode, growth, and developmental outcomes in infants with complex congenital heart disease at 6 and 12 months of age. J Pediatr.

[57] Knirsch W, Zingg W, Bernet V, Balmer C, Dimitropoulos A, Pretre R, Bauersfeld U, Latal B (2010). Determinants of body weight gain and association with neurodevelopmental outcome in infants operated for congenital heart disease. Interact Cardiovasc Thorac Surg.

[58] Heye KN, Rousson V, Knirsch W, Beck I, Liamlahi R, Bernet V, Dave H, Latal B, Heart, Brain Research G (2019). Growth and intellectual abilities of six-year-old children with congenital heart disease. J Pediatr.

[59] Mehta NM, Corkins MR, Lyman B, Malone A, Goday PS, Carney LN, Monczka JL, Plogsted SW, Schwenk WF, American Society for P, Enteral Nutrition Board of D (2013). Defining pediatric malnutrition: a paradigm shift toward etiology-related definitions. JPEN J Parenter Enteral Nutr.

[60] Koletzko B, Goulet O, Jochum F, Shamir R (2017). Use of parenteral nutrition in the pediatric ICU: should we panic because of PEPaNIC?. Curr Opin Clin Nutr Metab Care.

[61] Verlinden I, Coppens G, Vanhorebeek I, Guiza F, Derese I, Wouters PJ, Joosten KF, Verbruggen SC, Van den Berghe G (2023). Long-term impact of paediatric critical illness on the difference between epigenetic and chronological age in relation to physical growth. Clin Epigenetics.

[62] Manning JC, Pinto NP, Rennick JE, Colville G, Curley MAQ (2018). Conceptualizing post intensive care syndrome in children-the PICS-p framework. Pediatr Crit Care Med.

[63] Watson RS, Choong K, Colville G, Crow S, Dervan LA, Hopkins RO, Knoester H, Pollack MM, Rennick J, Curley MAQ (2018). Life after critical illness in children-toward an understanding of pediatric post-intensive care syndrome. J Pediatr.

[64] Verlinden I, Guiza F, Dulfer K, Van Cleemput H, Wouters PJ, Guerra GG, Joosten KF, Verbruggen SC, Vanhorebeek I, Van den Berghe G (2022). Physical, emotional/behavioral, and neurocognitive developmental outcomes from 2 to 4 years after PICU admission: a secondary analysis of the early versus late parenteral nutrition randomized controlled trial cohort. Pediatr Crit Care Med.

[65] Miller TA, Zak V, Shrader P, Ravishankar C, Pemberton VL, Newburger JW, Shillingford AJ, Dagincourt N, Cnota JF, Lambert LM, Sananes R, Richmond ME, Hsu DT, Miller SG, Zyblewski SC, Williams RV, Pediatric Heart Network I (2016). Growth asymmetry, head circumference, and neurodevelopmental outcomes in infants with single ventricles. J Pediatr.

