# Supplementary material for: Early weight measures and long-term neuropsychological outcome of critically ill neonates and infants: a secondary analysis of the PEPaNIC trial
Source: Eur J Pediatr. 2023 Nov 11;183(2):649–61. doi: 10.1007/s00431-023-05298-1 (PMC10912138; doi:10.1007/s00431-023-05298-1)
Supplement: Supplementary file 1 — Supplementary file1 (DOCX 480 KB) [file 431_2023_5298_MOESM1_ESM.docx]

# Supplementary files

## Supplementary appendix

*Appendix S1*. Summary of the PEPaNIC trial

*Appendix S2*. Definition of “Syndrome”

## *Appendix S3.* Statistical analysis

## *Appendix S4.* Definition of educational and occupational level of parents

## Supplementary appendix tables

*Table S1*. Comparison of characteristics of infants admitted to the Rotterdam PICU included and not included in analyses

*Table S2*. Weight measurements during hospital admission and at follow-up

*Table S3*. Change in weight during hospital admission

*Table S4*. Change in weight Z-score during different time periods - with paired comparisons

*Table S5*. Difference in weight Z-score between subgroups of patients

*Table S6*. Associations between growth per week in kilograms during hospital admission and outcomes at four year follow-up

*Table S7*. Weight for (gestational) age Z-score at PICU admission and outcomes at four year follow-up

*Table S8*. Weight for (gestational) age Z-score at hospital discharge and outcomes at four year follow-up

## Supplementary appendix figure

*Figure S1*. Correlation plot of the weight measurements at different time points

## *Figure S2 - Correlation plot of anthropometric and neuropsychological outcomes at 4-years follow-up*

## Appendix S1. Summary of the PEPaNIC trial

The PEPaNIC trial was conducted at the University Hospitals Leuven, Belgium; Erasmus MC – Sophia Children’s Hospital, Rotterdam, the Netherlands; and Stollery Children’s Hospital, Edmonton, AB, Canada. The study included 1440 critically ill infants and children admitted to the paediatric intensive care units of the participating centres between 2012 and 2015. In short, after obtaining parental consent for participation in this trial and long-term follow-up, participants were randomly assigned to receive early or late parenteral nutrition (PN), to investigate whether late PN was clinically superior to early PN. When randomised to the early PN strategy, PN was initiated within 24 hours of admission to the PICU to supplement enteral nutrition when enteral intake was less than 80% of targeted calories per age and weight category. Those randomised to late PN did not receive parenteral nutrition for up to one week in the PICU. Thereafter, PN was only initiated when enteral intake was still less than 80% of age- and weight specific targets (32).

Four years after PICU admission and initial participation in the PEPaNIC trial, survival status was assessed by reviewing hospital notes, obtained through the national register, or through contact with the general practitioner or referring paediatrician. After receiving a standardised patient information letter, survivors and parents or caregivers were contacted by telephone to obtain consent for scheduling an appointment for medical and neurocognitive assessment, either at the hospital or at the patient’s home. For patients who could not be reached by telephone, survival status was reassessed at the end of the study (7). The institutional review boards at each participating site approved this follow-up study (ML8052; NL49708.078; Pro00038098).

## Appendix S2. Definition of “Syndrome”

A pre-randomisation syndrome or illness a priori defined as affecting or possibly affecting neurocognitive development, and which is subdivided in the following categories (7, 8):

- Genetically confirmed syndrome or pathogenic chromosomal abnormality
- Clearly defined syndrome, association or malformation without (identified) genetic aberration
- Polymalformative syndrome of unknown aetiology
- Clear auditory or visual impairment without specified syndrome
- Congenital hypothyroidism due to thyroid agenesis
- Brain tumor or tumor with intracranial metastatic disease
- Paediatric psychiatric disorder (e.g., autism spectrum disorder, (treatment for) attention deficit hyperactivity disorder)
- Severe medical disorder, not primarily neurologic, but suspected to alter psychomotor and/or mental performance
- Severe neonatal problem (e.g. severe asphyxia)
- Severe craniocerebral trauma or near-drowning
- Severe infectious encephalitis or drug-induced encephalopathy
- Infectious meningitis, encephalitis or Guillain-Barré
- Resuscitation and/or need for extracorporeal membrane oxygenation prior to randomisation
- Severe convulsions or stroke prior to randomisation

## Appendix S3. Statistical analysis

*Missing data*

## Inability to fully complete the cognitive test battery could indicate poor cognitive function and thus introduce bias. Therefore, missing values were imputed by chained equations, with use of all available data for each individual. Imputation of data for age-specific tests was only done within the respective age group. Bias and instability of the imputation model was minimised by only including outcomes with no more than 30% missing data. The number of imputation models was set at 31 to avoid the loss of statistical power (7). The imputation was not re-done for these analyses.

##

## Appendix S4. Definition of educational and occupational level of parents (7, 8)(7, 8)

Educational level of parents

The education level is the average of the paternal and maternal educational level, and calculated based upon the 3-point scale subdivisions as made by the Centraal Bureau voor de Statistiek (The Netherlands; statline.cbs.nl): Low (=1), middle (=2) and high (=3) educational level

Occupational level of parents

The occupation level is the average of the paternal and maternal occupation level, which is calculated based upon the International Isco System 4-point scale for professions (http://www.ilo.org/public/english/bureau/stat/isco/). In case one of the parents filled in two jobs in the questionnaire, the highest Isco code level was used. In case “unemployed”, “disabled”, “student”, or “housewife/houseman” was filled in, an Isco code level of 1 was given to that parent. When the parents described their profession as “employee”, “worker”, “liberal profession”, or “retired”, they were given an Isco code level of 2.

## Table S1. Comparison of characteristics of infants admitted to the Rotterdam PICU included and not included in analyses

| Table S1 – Comparison of characteristics of infants admitted to the Rotterdam PICU included and not included in analyses | | | |
| --- | --- | --- | --- |
|  | Included in the analyses (N=121) | Not included in the analyses (N=188) | p value |
| Randomisation strategy |  |  | 0.627 |
| Early PN | 59 (48.8%) | 97 (51.6%) |  |
| Late PN | 62 (51.2%) | 91 (48.4%) |  |
| Sex |  |  | 0.269 |
| Male | 67 (55.4%) | 116 (61.7%) |  |
| Female | 54 (44.6%) | 72 (38.3%) |  |
| Age at admission (d) | 21.00 (1.00, 91.00) | 38.00 (2.00, 116.75) | 0.093 |
| Neonate at admission | 65 (53.7%) | 83 (44.1%) | 0.100 |
| Neonate (<7 days) at admission | 47 (38.8%) | 62 (33.0%) | 0.292 |
| PELOD score | 12.0 (3.0, 12.0) | 12.0 (11.0, 21.0) | 0.109 |
| PIM3 score |  |  | 0.180 |
| Number of observations | 120 | 33 |  |
| Median (IQR) | -3.7 (-4.4, -2.4) | -3.4 (-4.2, -2.1) |  |
| Elective or urgent admission |  |  | 0.803 |
| Elective | 30 (24.8%) | 49 (26.1%) |  |
| Urgent | 91 (75.2%) | 139 (73.9%) |  |
| Diagnostic group; cardiac surgery, other surgery versus medical |  |  | 0.175 |
| Surgical - Cardiac | 27 (22.3%) | 33 (17.6%) |  |
| Surgical - Other | 49 (40.5%) | 65 (34.6%) |  |
| Medical | 45 (37.2%) | 90 (47.9%) |  |
| STRONGkids risk category |  |  | 0.413 |
| Medium risk | 87 (71.9%) | 143 (76.1%) |  |
| High risk | 34 (28.1%) | 45 (23.9%) |  |
| Weight at admission (kg) | 3.62 (3.20, 4.65) | 3.90 (3.00, 5.62) | 0.627 |
| Weight at admission Z-score |  |  | 0.104 |
| Number of observations | 121 | 184 |  |
| Mean (SD) | -0.79 (1.63) | -1.12 (1.74) |  |
| Malnourished at admission | 29 (24.0%) | 44 (23.9%) | 0.991 |
| Duration of PICU stay (d) | 6.00 (3.00, 11.00) | 5.00 (2.00, 10.00) | 0.284 |
| Duration of hospital stay (d) | 13.00 (8.00, 29.00) | 12.00 (6.00, 24.00) | 0.048 |
| Groups were compared using a two-sample t-test, Kruskal-Wallis test or chi-square test. PICU = paediatric intensive care unit. PN = parenteral nutrition. PeLOD = paediatric logistic organ dysfunction score. PIM3 = paediatric index of mortality 3 score. STRONGkids = Screening Tool for Risk on Nutritional Status and Growth. ^^^PeLOD scores range from 0 to 71, with higher scores indicating more severe illness. ^^^^ Higher PIM3 scores indicate a higher risk of mortality. ^^^^^ STRONGkids scores range from 0 to 5, with a score of 0 indicating a low risk of malnutrition, a score of 1 to 3 indicating a medium risk and a score of 4 to 5 indicating a high risk. | | | |

## Table S2. Weight measurements during hospital admission and at follow-up

| Table S2 – Weight measurements during hospital admission and at follow-up | | | | | |
| --- | --- | --- | --- | --- | --- |
|  | PICU admissionN = 121 | PICU dischargeN = 121 | Hospital dischargeN = 121 | Two-year follow-upN = 105 | Four-year follow-upN = 121 |
| Age in days or years^*^, median (IQR) | 21.00 (1.00, 91.00) | 31.00 (11.00, 102.00) | 50.00 (21.00, 124.00) | 2.56 (2.52, 2.62) | 4.37 (4.22, 4.52) |
| Weight (kg), median (IQR) | 3.62 (3.20, 4.65) | 3.73 (3.24, 4.91) | 3.99 (3.40, 5.04) | 13.5 (12.4, 14.8) | 17.3 (15.5, 18.7) |
| Number of observations | 121 | 121 | 121 | 95 | 120 |
| Weight Z-score, mean (SD) | -0.79 (1.63)^****^ | -1.08 (1.57)^****^ | -1.25 (1.48)^****^ | 0.24 (1.11)^*^ | 0.11 (1.07)^ns^ |
| Malnourished (weight Z-score < -2), N (%) | 29 (24.0%) | 31 (25.6%) | 34 (28.1%) | 4 (3.8%) | 10 (8.3%) |
| **Number of observations** | 121 | 121 | 121 | 105 | 121 |
| PICU = paediatric intensive care unit, kg = kilograms. ^*^ Age is given in days for PICU admission, PICU discharge and hospital discharge and in years for two- and four-year follow-up | | | | | |
| ns: p > 0.05; * p ≤ 0.05; ** p ≤ 0.01; *** p ≤ 0.001; **** p ≤ 0.0001Two sided t-test comparing study group mean to population mean (0 with standard deviation 1) p-values adjusted using FDR correction for multiple testing | | | | | |

## Table S3. Change in weight during hospital admission

| Table S3 – Change in weight during hospital admission | | | |
| --- | --- | --- | --- |
|  | PICU admission – PICU dischargeN = 121 | PICU discharge – hospital dischargeN = 121 | PICU admission - hospital dischargeN = 121 |
| LOS (days), median (IQR) | 6.00 (3.00, 11.00) | 8.00 (2.00, 18.00) | 13.00 (8.00, 29.00) |
| Change in weight (g), median (IQR) | 20 (-120, 220) | 185 (39, 405) | 215 (18, 590) |
| Growth per week (g), median (IQR) | 24 (-155, 175) | 125 (30, 179) | 73 (-11, 140) |
| Change in weight Z-score, mean (SD) | -0.29 (0.64) | -0.17 (0.72) | -0.46 (0.82) |
| Category of change in weight Z-score, N (%) |  |  |  |
| Decline in Z-score | 69 (57.0%) | 59 (48.8%) | 93 (76.9%) |
| No change in Z-score | 20 (16.5%) | 25 (20.7%) | 2 (1.7%) |
| Incline in Z-score | 32 (26.4%) | 37 (30.6%) | 26 (21.5%) |
| Change in weight per age group |  |  |  |
| 0 to 3 months (N = 91) |  |  |  |
| Age-appropriate growth per week (g) | 200 | 200 | 200 |
| Growth per week in PICU population (g), median (IQR) | 0 (-198, 140) | 152 (58, 193) | 81 (0, 149) |
| 3 to 6 months (N = 19) |  |  |  |
| Age-appropriate growth per week (g) | 130 | 130 | 130 |
| Growth per week in PICU population (g), median (IQR) | 175 (60, 306) | 64 (-202, 132) | 72 (-26, 120) |
| 6 to 9 months (N = 5) |  |  |  |
| Age-appropriate growth per week (g) | 85 | 85 | 85 |
| Growth per week in PICU population (g), median (IQR) | 0 (0, 210) | -38 (-119, 16) | 0 (0, 13) |
| 9 to 12 months (N = 6) |  |  |  |
| Age-appropriate growth per week (g) | 75 | 75 | 75 |
| Growth per week in PICU population (g), median (IQR) | -152 (-336, -108) | 105 (-24, 149) | 1 (-96, 71) |
| LOS = length of stay, PICU = paediatric intensive care unit | | | |

## Table S4. Change in weight Z-score during different time periods - with paired comparisons

| Table S4 – Change in weight Z-score during different time periods – with paired comparisons | | | | | | | | | | | | |
| --- | --- | --- | --- | --- | --- | --- | --- | --- | --- | --- | --- | --- |
| End of period → | PICU discharge | | | Hospital discharge | | | Two-year follow-up | | | Four-year follow-up | | |
| Start of period ↓ | Difference (mean (SD)) | p-value | Adjusted p-value* | Difference (mean (SD)) | p-value | Adjusted p-value* | Difference (mean (SD)) | p-value | Adjusted p-value* | Difference (mean (SD)) | p-value | Adjusted p-value* |
| PICU admission | -0.29 (0.64) | <0.0001 | <0.0001 | -0.46 (0.82) | <0.0001 | <0.0001 | 1.06 (1.61) | <0.0001 | <0.0001 | 0.90 (1.68) | <0.0001 | <0.0001 |
| PICU discharge |  |  |  | -0.17 (0.72) | 0.010 | 0.012 | 1.35 (1.55) | <0.0001 | <0.0001 | 1.19 (1.62) | <0.0001 | <0.0001 |
| Hospital discharge |  |  |  |  |  |  | 1.50 (1.48) | <0.0001 | <0.0001 | 1.36 (1.54) | <0.0001 | <0.0001 |
| Two-year follow-up |  |  |  |  |  |  |  |  |  | -0.20 (0.94) | 0.032 | 0.032 |
| *p-values adjusted using FDR correction for multiple testing. PICU = paediatric intensive care unit | | | | | | | | | | | | |

## Table S5. Difference in weight Z-score between subgroups of patients

| Table S5 – Difference in weight Z-score between subgroups of patients | | | | | | | | | | |
| --- | --- | --- | --- | --- | --- | --- | --- | --- | --- | --- |
|  | Diagnostic group | | | | | | Age group | | | |
| Measurement time ↓ | Surgical – Cardiac | Surgical – Other | | Medical | p-value |  | Neonate (age <28 days at admission) | Non-neonate | p-value | Adjusted p-value* |
|  | mean (SD) | mean (SD) | | mean (SD) |  |  | mean (SD) | mean (SD) |  |  |
| PICU admission | -1.16 (1.63) | -0.52 (1.43) | | -0.87 (1.81) | 0.236 |  | -0.27 (1.12) | -1.41 (1.91) | 0.0002 | 0.001 |
| PICU discharge | -1.45 (1.55) | -0.79 (1.52) | | -1.17 (1.63) | 0.194 |  | -0.61 (1.20) | -1.63 (1.78) | 0.0005 | 0.001 |
| Hospital discharge | -1.53 (1.44) | -1.10 (1.49) | | -1.26 (1.49) | 0.476 |  | -0.96 (1.16) | -1.59 (1.72) | 0.022 | 0.037 |
| Two-year follow-up | 0.16 (1.26) | 0.08 (1.03) | | 0.44 (1.09) | 0.319 |  | 0.34 (1.15) | 0.13 (1.07) | 0.333 | 0.416 |
| Four-year follow-up | -0.02 (0.94) | 0.04 (1.15) | | 0.26 (1.07) | 0.469 |  | 0.16 (1.16) | 0.05 (0.97) | 0.583 | 0.583 |
|  | Nutritional status at PICU admission | | | | | | Nutritional status at hospital discharge | | | |
| Measurement time ↓ | Malnutrition (weight Z-score < -2) | | No malnutrition | | p-value | Adjusted p-value* | Malnutrition (weight Z-score < -2) | No malnutrition | p-value | Adjusted p-value* |
|  | mean (SD) | | mean (SD) | |  |  | mean (SD) | mean (SD) |  |  |
| PICU admission | -3.06 (0.89) | | -0.08 (1.05) | | <0.0001 | <0.0001 | -2.56 (1.25) | -0.07 (1.19) | <0.0001 | <0.0001 |
| PICU discharge | -3.14 (0.93) | | -0.43 (1.11) | | <0.0001 | <0.0001 | -2.80 (1.23) | -0.40 (1.15) | <0.0001 | <0.0001 |
| Hospital discharge | -3.03 (1.08) | | -0.70 (1.09) | | <0.0001 | <0.0001 | -3.05 (0.93) | -0.56 (0.98) | <0.0001 | <0.0001 |
| Two-year follow-up | -0.16 (1.28) | | 0.37 (1.02) | | 0.060 | 0.060 | -0.42 (1.11) | 0.47 (1.04) | 0.001 | 0.001 |
| Four-year follow-up | -0.23 (0.98) | | 0.21 (1.08) | | 0.044 | 0.054 | -0.30 (1.23) | 0.25 (0.98) | 0.054 | 0.054 |
| *p-values adjusted using FDR correction for multiple testing. PICU = paediatric intensive care unit | | | | | | | | | | |

## Table S6. – Associations between growth per week in kilograms during hospital admission and outcomes at four year follow-up

|  | Number (%) of observations | Pooled outcome | Univariable analysis | | Multivariable analysis^a^ | |
| --- | --- | --- | --- | --- | --- | --- |
|  | Total N = 121 | Mean (SD) | β-estimate (95%CI) | Adjusted p-value | β-estimate (95%CI) | Adjusted p-value |
| Anthropometrics |  |  |  |  |  |  |
| Height Z-score^^^ | 121 (100%) | 0.16 (1.07) | -1.09 (-2.20, 0.01) | 0.420 | -0.77 (-1.98, 0.44) | 0.808 |
| Weight Z-score^^^ | 121 (100%) | 0.11 (1.07) | -0.73 (-1.84, 0.39) | 0.575 | -0.26 (-1.49, 0.96) | 0.808 |
| BMI Z-score^^^ | 120 (99.2%) | 0.03 (1.2) | 0.19 (-1.07, 1.45) | 0.835 | 0.55 (-0.79, 1.88) | 0.808 |
| Executive functions (BRIEF^#)^ |  |  |  |  |  |  |
| Emotional control | 121 (100%) | 50.1 (10.5) | 1.71 (-9.36, 12.78) | 0.835 | 2.74 (-9.74, 15.22) | 0.808 |
| Flexibility | 121 (100%) | 50.5 (11.6) | 1.69 (-10.53, 13.92) | 0.835 | 3.46 (-10.35, 17.27) | 0.808 |
| Inhibition | 121 (100%) | 50.5 (11.5) | 5.53 (-6.55, 17.62) | 0.678 | 7.09 (-6.39, 20.56) | 0.808 |
| Working memory | 121 (100%) | 51.7 (11.9) | 4.37 (-8.08, 16.82) | 0.718 | 3.01 (-10.92, 16.94) | 0.808 |
| Meta-cognition index | 121 (100%) | 51.0 (11.3) | 5.28 (-6.57, 17.14) | 0.678 | 4.40 (-8.89, 17.69) | 0.808 |
| Planning and organisation | 121 (100%) | 50.4 (10.2) | 5.76 (-4.92, 16.44) | 0.654 | 5.35 (-6.73, 17.42) | 0.808 |
| Total score | 121 (100%) | 50.7 (11.9) | 4.88 (-7.62, 17.39) | 0.689 | 5.60 (-8.43, 19.63) | 0.808 |
| Emotional and behavioral problems (CBCL^#)^ |  |  |  |  |  |  |
| Externalising problems | 121 (100%) | 47.3 (10.2) | 6.50 (-4.16, 17.16) | 0.575 | 3.89 (-7.54, 15.31) | 0.808 |
| Internalising problems | 121 (100%) | 49.4 (10.6) | 5.45 (-5.66, 16.57) | 0.678 | 2.45 (-9.64, 14.55) | 0.808 |
| Total | 121 (100%) | 48.0 (10.7) | 8.02 (-3.16, 19.20) | 0.564 | 4.58 (-7.39, 16.55) | 0.808 |
| IQ^~^ |  |  |  |  |  |  |
| Performal IQ | 121 (100%) | 92.3 (13.1) | -2.61 (-16.38, 11.16) | 0.835 | -2.48 (-17.45, 12.49) | 0.808 |
| Verbal IQ | 121 (100%) | 92.7 (15.0) | 0.20 (-15.53, 15.94) | 0.980 | -1.71 (-18.59, 15.17) | 0.841 |
| Total IQ | 121 (100%) | 96.6 (16.8) | 4.79 (-12.83, 22.41) | 0.821 | 2.11 (-16.87, 21.10) | 0.841 |
| Motor coordination^~^ |  |  |  |  |  |  |
| Number of unimanual taps (left hand) | 101 (83.5%) | 23.4 (6.1) | -2.71 (-9.29, 3.88) | 0.689 | -2.65 (-10.14, 4.84) | 0.808 |
| Number of unimanual taps (right hand) | 101 (83.5%) | 25.7 (6.6) | -0.91 (-8.05, 6.24) | 0.835 | -1.42 (-8.99, 6.14) | 0.808 |
| Number of valid alternating taps | 101 (83.5%) | 11.8 (8.6) | -6.69 (-15.98, 2.60) | 0.564 | -6.99 (-17.32, 3.33) | 0.808 |
| Number of valid synchronous taps | 101 (83.5%) | 7.5 (6.0) | -6.86 (-13.27, -0.46) | 0.420 | -7.54 (-14.79, -0.30) | 0.808 |
| Alertness^#^ |  |  |  |  |  |  |
| Reaction time left hand (Z-score) | 101 (83.5%) | 1.6 (1.4) | 1.33 (-0.18, 2.85) | 0.420 | 1.21 (-0.51, 2.93) | 0.808 |
| Within subject SD of repeated tests (Z-score) | 101 (83.5%) | 2.6 (1.5) | 1.60 (-0.04, 3.23) | 0.420 | 1.49 (-0.36, 3.34) | 0.808 |
| Reaction time right hand (Z-score) | 101 (83.5%) | 3.0 (1.4) | 1.37 (-0.18, 2.92) | 0.420 | 1.11 (-0.64, 2.86) | 0.808 |
| Within subject SD of repeated tests (Z-score) | 101 (83.5%) | 2.3 (1.2) | 0.85 (-0.50, 2.20) | 0.575 | 0.60 (-0.94, 2.14) | 0.808 |
| Visual Motor-Integration scale^~^ | 121 (100%) | 9.8 (2.2) | 0.44 (-1.89, 2.77) | 0.835 | 0.47 (-2.06, 2.99) | 0.808 |
| Results are presented in numbers with proportions (%), mean (SD), β-estimates (95%CI). BMI = body-mass index. BRIEF = Behaviour Rating Inventory of Executive Function (parent-reported). CBCL = Child Behaviour Checklist (parent-reported). IQ = intelligence quotient. SES = socio-economic status. ^a^ Adjusted for gender, diagnostic group, age group at admission, a predefined syndrome, PIM3 score, parental smoking behaviour before admission to the PICU, and occupational level of the parents or caregivers. ^^^ Age-sex-adjusted Z-scores were calculated with the use of reference data from Fenton- and nationally available growth charts. ^#^ Higher scores reflect worse performance. ^~^ Higher scores reflect better performance.p-values adjusted using FDR correction for multiple testing | | | | | | |

## Table S7. Weight for (gestational) age Z-score at PICU admission and outcomes at four year follow-up

|  | Number (%) of observations | Pooled outcome | Univariable analysis | | Multivariable analysis^a^ | |
| --- | --- | --- | --- | --- | --- | --- |
|  | Total N = 121 | Mean (SD) | β-estimate (95%CI) | Adjusted p-value | β-estimate (95%CI) | Adjusted p-value |
| Anthropometrics |  |  |  |  |  |  |
| Height Z-score^^^ | 121 (100%) | 0.16 (1.07) | 0.24 (0.13, 0.35) | 0.001 | 0.22 (0.10, 0.35) | 0.014 |
| Weight Z-score^^^ | 121 (100%) | 0.11 (1.07) | 0.19 (0.07, 0.30) | 0.02 | 0.20 (0.08, 0.33) | 0.022 |
| BMI Z-score^^^ | 120 (99.2%) | 0.03 (1.20) | 0.08 (-0.05, 0.22) | 0.584 | 0.12 (-0.03, 0.26) | 0.689 |
| BRIEF^#^ |  |  |  |  |  |  |
| Emotional control | 121 (100%) | 50.1 (10.5) | -0.60 (-1.77, 0.56) | 0.593 | -0.22 (-1.57, 1.13) | 0.829 |
| Flexibility | 121 (100%) | 50.5 (11.6) | -0.11 (-1.40, 1.19) | 0.922 | 0.33 (-1.16, 1.83) | 0.829 |
| Inhibition | 121 (100%) | 50.5 (11.5) | -1.24 (-2.51, 0.02) | 0.338 | -0.90 (-2.35, 0.56) | 0.801 |
| Working memory | 121 (100%) | 51.7 (11.9) | -0.43 (-1.75, 0.89) | 0.68 | -0.35 (-1.85, 1.16) | 0.829 |
| Meta-cognition index | 121 (100%) | 51.0 (11.3) | -0.38 (-1.63, 0.88) | 0.695 | -0.35 (-1.79, 1.09) | 0.829 |
| Planning and organisation | 121 (100%) | 50.4 (10.2) | -0.41 (-1.55, 0.72) | 0.68 | -0.40 (-1.70, 0.91) | 0.829 |
| Total score | 121 (100%) | 50.7 (11.9) | -0.75 (-2.07, 0.57) | 0.584 | -0.45 (-1.97, 1.07) | 0.829 |
| CBCL^#^ |  |  |  |  |  |  |
| Externalising problems | 121 (100%) | 47.3 (10.2) | -1.39 (-2.50, -0.28) | 0.122 | -1.11 (-2.33, 0.11) | 0.609 |
| Internalising problems | 121 (100%) | 49.4 (10.6) | -0.64 (-1.82, 0.53) | 0.584 | -0.50 (-1.81, 0.80) | 0.829 |
| Total | 121 (100%) | 48.0 (10.7) | -1.09 (-2.27, 0.09) | 0.347 | -0.92 (-2.21, 0.36) | 0.757 |
| IQ^~^ |  |  |  |  |  |  |
| Performal IQ | 121 (100%) | 92.3 (13.1) | 0.48 (-0.97, 1.94) | 0.68 | 0.11 (-1.51, 1.73) | 0.928 |
| Verbal IQ | 121 (100%) | 96.6 (16.8) | 1.39 (-0.46, 3.24) | 0.502 | 0.35 (-1.70, 2.40) | 0.829 |
| Total IQ | 121 (100%) | 92.7 (15.0) | 1.32 (-0.33, 2.97) | 0.483 | 0.44 (-1.38, 2.26) | 0.829 |
| Motor coordination^~^ |  |  |  |  |  |  |
| Number of unimanual taps (left hand) | 101 (83.5%) | 23.4 (6.1) | 0.25 (-0.47, 0.96) | 0.68 | 0.26 (-0.58, 1.11) | 0.829 |
| Number of unimanual taps (right hand) | 101 (83.5%) | 25.7 (6.6) | 0.48 (-0.28, 1.25) | 0.584 | 0.57 (-0.27, 1.41) | 0.757 |
| Number of valid alternating taps | 101 (83.5%) | 11.8 (8.6) | 0.60 (-0.41, 1.61) | 0.584 | 0.59 (-0.58, 1.75) | 0.829 |
| Number of valid synchronous taps | 101 (83.5%) | 7.5 (6.0) | 0.24 (-0.47, 0.95) | 0.68 | 0.43 (-0.39, 1.26) | 0.829 |
| Alertness^#^ |  |  |  |  |  |  |
| Reaction time left hand (Z-score) | 101 (83.5%) | 1.6 (1.4) | -0.02 (-0.19, 0.14) | 0.893 | 0.00 (-0.19, 0.20) | 0.997 |
| Within subject SD of repeated tests (Z-score) | 101 (83.5%) | 2.3 (1.2) | 0.01 (-0.14, 0.16) | 0.922 | 0.03 (-0.15, 0.20) | 0.829 |
| Reaction time right hand (Z-score) | 101 (83.5%) | 2.6 (1.5) | -0.06 (-0.24, 0.12) | 0.68 | -0.04 (-0.25, 0.17) | 0.829 |
| Within subject SD of repeated tests (Z-score) | 101 (83.5%) | 3.0 (1.4) | -0.03 (-0.20, 0.14) | 0.834 | -0.06 (-0.25, 0.14) | 0.829 |
| Visual Motor-Integration scale^~^ | 121 (100%) | 9.8 (2.2) | -0.01 (-0.26, 0.23) | 0.922 | -0.08 (-0.36, 0.19) | 0.829 |
| Results are presented in numbers with proportions (%), mean (SD), β-estimates (95%CI). BMI = body-mass index. BRIEF = Behaviour Rating Inventory of Executive Function. CBCL = Child Behaviour Checklist . IQ = intelligence quotient. SES = socio-economic status. ^a^ Adjusted for gender, diagnostic group, age group at admission, a predefined syndrome, PIM3 score, parental smoking behaviour before admission to the PICU, and occupational level of the parents or caregivers. ^^^ Age-sex-adjusted Z-scores were calculated with the use of reference data from Fenton- and nationally available growth charts. ^#^ Higher scores reflect worse performance. ^~^ Higher scores reflect better performance p-values adjusted using FDR correction for multiple testing | | | | | | |

## Table S8. Weight for (gestational) age Z-score at hospital discharge and outcomes at four year follow-up

| Table S8 – Weight for (gestational) age Z-score at hospital discharge and outcomes at four year follow-up | | | | | | |
| --- | --- | --- | --- | --- | --- | --- |
|  | Number (%) of observations | Pooled outcome | Univariable analysis | | Multivariable analysis^a^ | |
|  | Total N = 121 | Mean (SD) | β-estimate (95%CI) | Adjusted p-value | β-estimate (95%CI) | Adjusted p-value |
| Anthropometrics |  |  |  |  |  |  |
| Height Z-score^^^ | 121 (100%) | 0.16 (1.07) | 0.20 (0.07, 0.32) | 0.033 | 0.18 (0.04, 0.31) | 0.113 |
| Weight Z-score^^^ | 121 (100%) | 0.11 (1.07) | 0.22 (0.09, 0.34) | 0.022 | 0.24 (0.11, 0.38) | 0.014 |
| BMI Z-score^^^ | 120 (99.2%) | 0.03 (1.20) | 0.16 (0.02, 0.31) | 0.138 | 0.21 (0.06, 0.37) | 0.069 |
| BRIEF^#^ |  |  |  |  |  |  |
| Emotional control | 121 (100%) | 50.1 (10.5) | -0.74 (-2.03, 0.56) | 0.503 | -0.31 (-1.76, 1.15) | 0.912 |
| Flexibility | 121 (100%) | 50.5 (11.6) | -0.44 (-1.87, 0.99) | 0.795 | -0.05 (-1.66, 1.56) | 0.977 |
| Inhibition | 121 (100%) | 50.5 (11.5) | -1.40 (-2.79, 0.00) | 0.18 | -0.93 (-2.49, 0.64) | 0.76 |
| Working memory | 121 (100%) | 51.7 (11.9) | -0.45 (-1.91, 1.01) | 0.795 | -0.27 (-1.89, 1.35) | 0.912 |
| Meta-cognition index | 121 (100%) | 51.0 (11.3) | -0.33 (-1.73, 1.06) | 0.795 | -0.21 (-1.76, 1.34) | 0.912 |
| Planning and organisation | 121 (100%) | 50.4 (10.2) | -0.31 (-1.57, 0.94) | 0.795 | -0.20 (-1.61, 1.21) | 0.912 |
| Total score | 121 (100%) | 50.7 (11.9) | -0.89 (-2.35, 0.57) | 0.479 | -0.51 (-2.14, 1.13) | 0.912 |
| CBCL^#^ |  |  |  |  |  |  |
| Externalising problems | 121 (100%) | 47.3 (10.2) | -1.57 (-2.79, -0.34) | 0.106 | -1.05 (-2.37, 0.27) | 0.525 |
| Internalising problems | 121 (100%) | 49.4 (10.6) | -1.14 (-2.44, 0.15) | 0.205 | -0.88 (-2.28, 0.52) | 0.76 |
| Total | 121 (100%) | 48.0 (10.7) | -1.48 (-2.77, -0.18) | 0.138 | -1.08 (-2.46, 0.31) | 0.525 |
| IQ^~^ |  |  |  |  |  |  |
| Performal IQ | 121 (100%) | 92.3 (13.1) | 1.06 (-0.54, 2.67) | 0.434 | 0.72 (-1.02, 2.46) | 0.912 |
| Verbal IQ | 121 (100%) | 96.6 (16.8) | 1.81 (-0.23, 3.86) | 0.205 | 0.80 (-1.41, 3.00) | 0.912 |
| Total IQ | 121 (100%) | 92.7 (15.0) | 1.93 (0.12, 3.74) | 0.154 | 1.07 (-0.88, 3.03) | 0.778 |
| Motor coordination^~^ |  |  |  |  |  |  |
| Number of unimanual taps (left hand) | 101 (83.5%) | 23.4 (6.1) | 0.20 (-0.61, 1.01) | 0.795 | 0.16 (-0.77, 1.09) | 0.912 |
| Number of unimanual taps (right hand) | 101 (83.5%) | 25.7 (6.6) | 0.81 (-0.05, 1.68) | 0.205 | 0.85 (-0.07, 1.76) | 0.435 |
| Number of valid alternating taps | 101 (83.5%) | 11.8 (8.6) | 0.16 (-1.00, 1.31) | 0.898 | -0.02 (-1.31, 1.27) | 0.977 |
| Number of valid synchronous taps | 101 (83.5%) | 7.5 (6.0) | 0.003 (-0.80, 0.81) | 0.994 | 0.11 (-0.81, 1.02) | 0.912 |
| Alertness^#^ |  |  |  |  |  |  |
| Reaction time left hand (Z-score) | 101 (83.5%) | 1.6 (1.4) | -0.002 (-0.19, 0.19) | 0.994 | 0.04 (-0.17, 0.26) | 0.912 |
| Within subject SD of repeated tests (Z-score) | 101 (83.5%) | 2.3 (1.2) | 0.03 (-0.14, 0.19) | 0.898 | 0.07 (-0.12, 0.26) | 0.912 |
| Reaction time right hand (Z-score) | 101 (83.5%) | 2.6 (1.5) | -0.09 (-0.29, 0.12) | 0.692 | -0.02 (-0.26, 0.21) | 0.912 |
| Within subject SD of repeated tests (Z-score) | 101 (83.5%) | 3.0 (1.4) | -0.05 (-0.24, 0.14) | 0.795 | -0.03 (-0.25, 0.19) | 0.912 |
| Visual Motor-Integration scale^~^ | 121 (100%) | 9.8 (2.2) | 0.01 (-0.26, 0.29) | 0.994 | -0.05 (-0.34, 0.24) | 0.912 |
| Results are presented in numbers with proportions (%), mean (SD), β-estimates (95%CI). BMI = body-mass index. BRIEF = Behaviour Rating Inventory of Executive Function. CBCL = Child Behaviour Checklist . IQ = intelligence quotient. SES = socio-economic status. ^a^ Adjusted for gender, diagnostic group, age group at admission, a predefined syndrome, PIM3 score, parental smoking behaviour before admission to the PICU, and occupational level of the parents or caregivers. ^^^ Age-sex-adjusted Z-scores were calculated with the use of reference data from Fenton- and nationally available growth charts. ^#^ Higher scores reflect worse performance. ^~^ Higher scores reflect better performance p-values adjusted using FDR correction for multiple testing | | | | | | |

## Figure S1. Correlation plot of the weight measurements at different time points

| Figure S1 - Correlation plot of the weight measurements at different time points |
| --- |
| 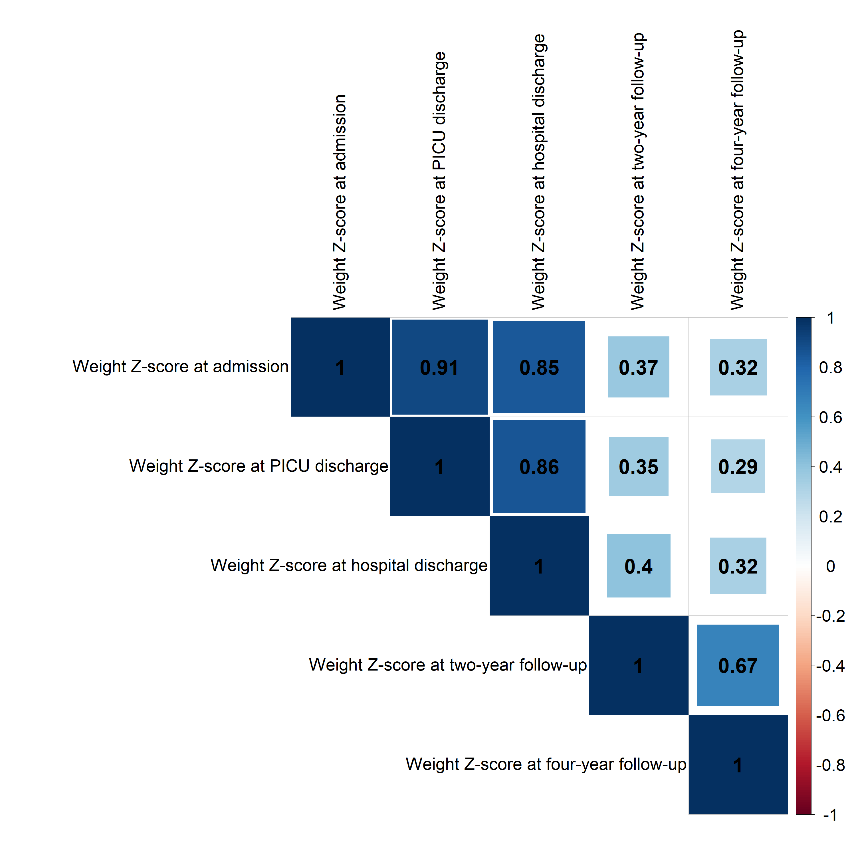 |
| Visual representation of the correlation between weight measurements taken at different time points. Blue shades represent a positive correlation, red shades represent a negative correlation. The darker the color, the stronger the correlation. Calculated Spearman’s rho are depicted in every square. |

## Figure S2 - Correlation plot of anthropometric and neuropsychological outcomes at 4-years follow-up

|  |
| --- |
| 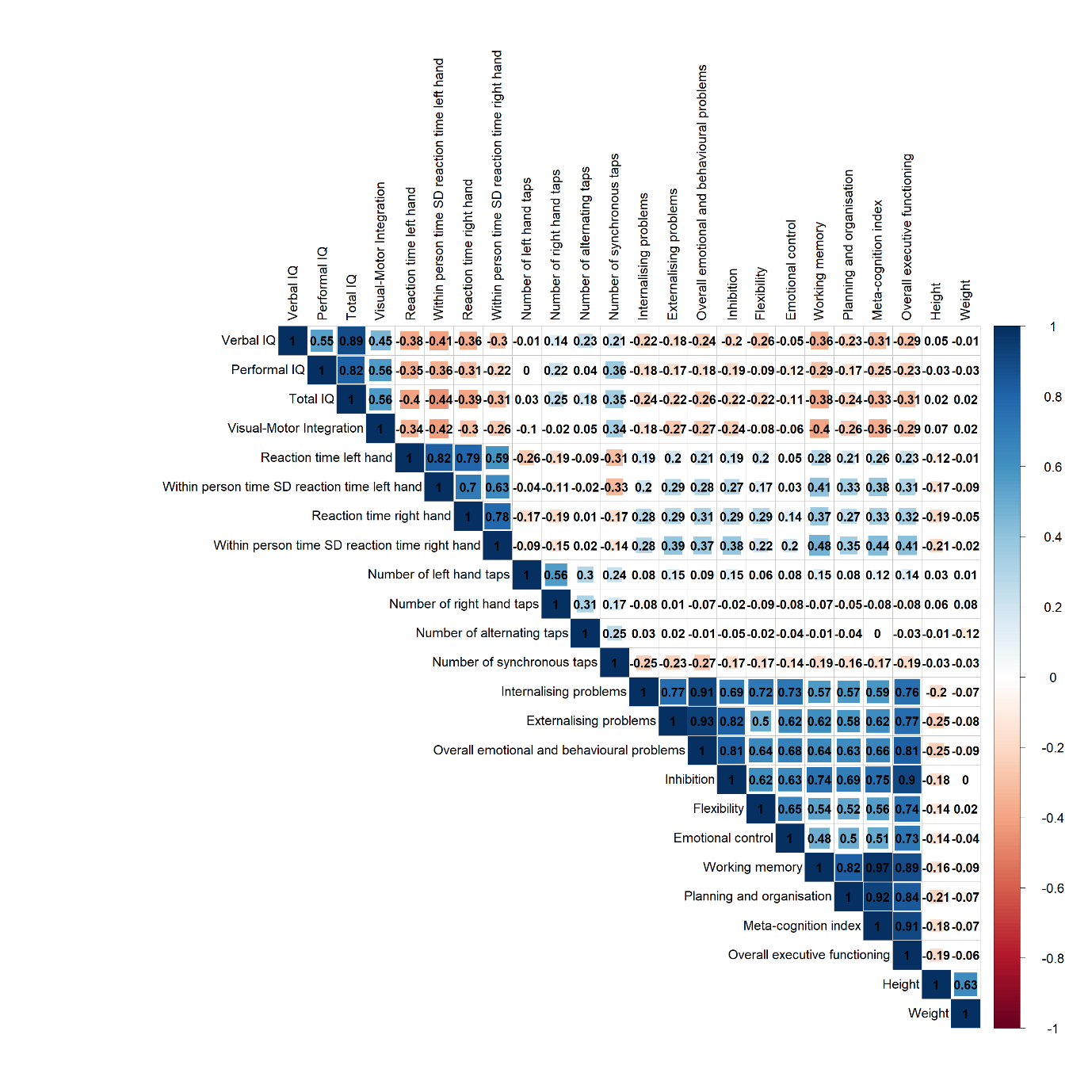 |
| Visual representation of the correlation between outcome measures assessed at four-year follow-up. Blue shades represent a positive correlation, red shades represent a negative correlation. The darker the color, the stronger the correlation. Calculated Spearman’s rho are depicted in every square. |
